# The impact of the COVID-19 pandemic on *Staphylococcus aureus* infections in pediatric patients admitted with community acquired pneumonia

**DOI:** 10.1038/s41598-024-66071-4

**Published:** 2024-07-08

**Authors:** Ling Ai, Liang Fang, Chanjuan Zhou, Beizhong Liu, Quan Yang, Fang Gong

**Affiliations:** 1 Department of General Practice, Yongchuan Hospital of Chongqing Medical University, No. 439, Xuanhua Street, Chongqing, 402160 China; 2 Department of Respiratory and Critical Care Medicine, The Affiliated Hospital of Southwest Medical University, Luzhou, 646000 Sichuan China; 3 Central Laboratory, Yongchuan Hospital of Chongqing Medical University, Chongqing, 402160 China; 4 Department of Neurology, Yongchuan Hospital of Chongqing Medical University, Chongqing, 402160 China; 5 Key Laboratory of Laboratory Medical Diagnostics, Ministry of Education,, Department of Laboratory Medicine, Chongqing Medical University, Chongqing, 400016 China; 6 Department of Radiology, Yongchuan Hospital of Chongqing Medical University, Chongqing, 402160 China; 7 Department of Pediatrics, Yongchuan Hospital of Chongqing Medical University, Chongqing, 402160 China

**Keywords:** Pediatrics, COVID-19, *Staphylococcus aureus*, Epidemiology, Antimicrobial resistance, Community acquired pneumonia, Infectious diseases, Epidemiology

## Abstract

The COVID-19 pandemic has significantly transformed the infection spectrum of various pathogens. This study aimed to evaluate the impact of the COVID-19 pandemic on *Staphylococcus aureus* (*S. aureus*) infections among pediatric patients with community acquired pneumonia (CAP). We retrospectively reviewed pediatric CAP admissions before (from 2018 to 2019) and during (from 2020 to 2022) the COVID-19 pandemic. The epidemiology and antimicrobial resistance (AMR) profiles of *S. aureus* isolates were examined to assess the pandemic’s effect. As a result, a total of 399 pediatric CAP patients with *S. aureus* infections were included. The positivity rate, gender, and age distribution of patients were similar across both periods. There was a marked reduction in respiratory co-infections with *Haemophilus influenzae* (*H. influenzae*) during the COVID-19 pandemic, compared to 2019. Additionally, there were significant changes in the resistance profiles of *S. aureus* isolates to various antibiotics. Resistance to oxacillin and tetracycline increased, whereas resistance to penicillin, gentamicin, and quinolones decreased. Notably, resistance to erythromycin significantly decreased in methicillin-resistant *S. aureus* (MRSA) strains. The number of *S. aureus* isolates, the proportion of viral co-infections, and the number of resistant strains typically peaked seasonally, primarily in the first or fourth quarters of 2018, 2019, and 2021. However, shifts in these patterns were noted in the first quarter of 2020 and the fourth quarter of 2022. These findings reveal that the COVID-19 pandemic has significantly altered the infection dynamics of *S. aureus* among pediatric CAP patients, as evidenced by changes in respiratory co-infections, AMR patterns, and seasonal trends.

## Introduction

Community acquired pneumonia (CAP) poses a high burden of morbidity and mortality, also leading to significant healthcare costs. The incidence of CAP varies by region, season, and demographic group. It primarily affects children under five and adults over sixty-five years old^1^. *Staphylococcus aureus* (*S. aureus*), one of the Gram-positive opportunistic pathogens, can lead to various illnesses, including pneumonia, sepsis, meningitis, and soft tissue and skin infections^2^. Beyond the hospital settings, community-acquired *S. aureus* plays a significant role in pneumonia cases^3^. The detection of *S. aureus* isolates with high antimicrobial resistance (AMR), especially methicillin-resistant *S. aureus* (MRSA), has intensified management challenges. *S. aureus* is among the top seven pathogens responsible for approximately 457,000 AMR-related deaths in 53 European countries, with MRSA being the predominant cause in 27 of these countries^4^. Continuous monitoring of *S. aureus* epidemiology and AMR trends is essential.

To control the spread of COVID-19, China implemented various non-pharmaceutical interventions (NPIs) from January 2020 to December 2022. These measures included universal symptom surveys, social distancing, cordon sanitaire, quarantine strategies, and transport restrictions^5^. During this period, the COVID-19 pandemic coincided with shifts in the epidemiology and AMR patterns of several human pathogens, including *S. aureus*. Although the incidence of *S. aureus* infections might have remained stable during the pandemic^6^, numerous studies indicate significant fluctuations in the number of *S. aureus* isolates due to pandemic-related changes^7,8^. Changes in antibiotic resistance rates and variations in sequence types and molecular characteristics of *S. aureus* have also been observed, differing from pre-pandemic patterns^9–11^. However, these studies often cover short periods within the pandemic and rarely focus on *S. aureus* strains from pediatric CAP patients.

This retrospective study aims to evaluate the changes in bacterial epidemiology and AMR patterns of *S. aureus* isolates in pediatric CAP patients during the COVID-19 pandemic. It is an analysis that includes patient data from 2018 to 2022, thus covering two years before and three years during the pandemic.

## Materials and methods

### Study population

This is a retrospective study conducted in Yongchuan Hospital of Chongqing Medical University, a tertiary hospital of southwest China, between January 2018 and December 2022. We identified pediatric patients with CAP through a comprehensive approach which included clinical manifestations, laboratory tests, and chest imaging. Expert radiologists evaluated chest imaging to identify findings indicative of pneumonia, such as infiltrates, consolidation, and other relevant abnormalities. The study enrolled patients aged between 1 month and 18 years. Exclusion criteria were: (1) Pneumonia occurred ≥ 48 h post admission, (2) Chest imaging findings of pleural effusion, lobar pneumonia, alveolar infiltrate, or interstitial infiltrate > 72 h after admission, (3) Lung interstitial or infiltrate changes attributed to atelectasis, pulmonary edema, or pulmonary tuberculosis, and (4) Patients without bacterial culture results or complete medical records. The Yongchuan Hospital’s ethics committee approved the study protocol (No. 2023-KeLunShen-76). All methods were carried out according to relevant regulations and guidelines.

### *S. aureus* strains identification and antimicrobial susceptibility testing

Sputum samples were collected for microbiological testing upon admission, following established clinical protocols. For patients unable to produce sputum, samples were obtained from the nasopharynx or through deep suction under negative pressure. The quality of sputum was considered adequate when it contained at least 25 leukocytes and no more than 10 epithelial cells were observed under low magnification. Samples were then cultured on blood, MacConkey, and chocolate agar plates, followed by incubation at 37 ℃ in a 5% CO2 environment for 18–24 h. *S. aureus* was identified by using the Vitek-2 Compact system (BioMérieux, France). Antimicrobial sensitivity was assessed using ATB identification cards, testing 15 antibiotics: oxacillin, penicillin, erythromycin, clindamycin, tetracycline, trimethoprim-sulfamethoxazole, gentamicin, ciprofloxacin, levofloxacin, moxifloxacin, rifampicin, quinupristin-dalfopristin, tigecycline, linezolid, and vancomycin. A cefoxitin screening test was also conducted. All tests adhered to Clinical and Laboratory Standards Institute (CLSI) guidelines, categorizing isolates as susceptible, intermediate, or resistant. MRSA status was determined based on the resistance to oxacillin or a positive cefoxitin screening. The study included only unique *S. aureus* strains, excluding repeated isolates from the same patient during a hospitalization episode. *S. aureus* ATCC 29213 served as the quality-control strain.

To differentiate between colonization and *S. aureus* as the causative agent of CAP, several diagnostic criteria were applied in clinical practice. To consider *S. aureus* as a causative agent rather than a colonizer, the presence of symptoms consistent with CAP, such as fever and cough, along with radiological evidence of pneumonia, was required. Additionally, high bacterial loads, determined through quantitative cultures when applicable, indicated infection rather than colonization. Elevated systemic markers of inflammation, such as C-reactive protein and procalcitonin, supporting the presence of an active infection, were considered alongside microbiological findings. The attending clinician’s judgment, based on the patient’s clinical manifestation and the course of the illness, also played a crucial role in distinguishing colonization from infection.

### Identification of respiratory co-infections

Our study examined respiratory co-infections involving bacteria, *Mycoplasma pneumoniae* (*M. pneumoniae*), and viruses among pediatric CAP patients infected with *S. aureus*. We specifically evaluated bacterial co-infections with *Streptococcus pneumoniae* (*S. pneumoniae*), *Haemophilus influenzae* (*H. influenzae*), and *Moraxella catarrhalis* (*M. catarrhalis*). Co-infections were confirmed when sputum specimens tested positive for both *S. aureus* and another bacterial species. To detect *M. pneumoniae* co-infections, serum was separated from venous blood samples. The presence of *M. pneumoniae* was determined by detecting Immunoglobulin M (IgM) antibodies in the serum using an indirect immunofluorescence assay (IFA) or a passive particle agglutination test (Fujirebio, Japan). An antibody titer of ≥ 1:160 in the passive agglutination test indicated an *M. pneumoniae* infection.

For detecting viral co-infections, nasopharyngeal swab or venous blood samples were collected from patients at admission. Our viral testing targeted five primary respiratory viruses: influenza virus A (IVA), influenza virus B (IVB), parainfluenza virus (PIV), respiratory syncytial virus (RSV), and adenovirus (ADV). Nasopharyngeal swab samples were analyzed using a multiplex direct immunofluorescence assay kit (Diagnostic Hybrids, Athens, Ohio, USA), adhering to established protocols. Serum IgM antibodies against these viruses were quantified using IFA for venous blood samples. Viral co-infections were identified based on positive results from either nasopharyngeal swab or serum samples.

### Statistical analysis

We assessed the normality of quantitative data using the Kolmogorov–Smirnov test. Data following a normal distribution were expressed as mean ± standard deviation (SD) and compared between groups using the Student’s t-test. For non-normally distributed data, medians and interquartile ranges were used, with group comparisons conducted using the Mann–Whitney U test. Categorical variables were compared using a two-tailed chi-square test, Fisher’s exact test, or Yates’ continuity corrected chi-square test, depending on the actual and theoretical frequencies. Co-infections were analyzed after excluding patients without corresponding pathogenic results. All statistical analyses were performed using GraphPad Prism 9.0 Software (GraphPad Software, Inc., San Diego, CA, USA). A *P*-value of less than 0.05 was considered statistically significant.

### Ethics approval

The study protocol was approved by the ethics committee of the Yongchuan Hospital of Chongqing Medical University (No. 2023-KeLunShen-76).

### Consent to participate

As a retrospective study, The Yongchuan Hospital’s ethics committee waived the need for informed consent.

## Results

### Impact of the COVID-19 pandemic on the positivity rates, demographic characteristics, and respiratory co-infections of *S. aureus*-associated CAP patients

This study included 6115 children admitted with CAP from January 2018 to December 2022. Of these, 5941 had respiratory specimens, specifically sputum, collected for bacterial culture upon admission, with 399 (6.7%) testing positive for *S. aureus*. Despite a decrease in the number of isolates from 2020 to 2022 during the COVID-19 pandemic, the positivity rate of *S. aureus* among those tested did not significantly change. The most common symptoms of *S. aureus*-associated CAP were cough (91.0%), wheezing (22.6%), vomiting (16.5%), and fever (15.5%). The cohort consisted of 233 (58.4%) males and 166 (41.6%) females, with a median age of 2 months. The distribution of *S. aureus* infections across different age groups was: 76.9% (307 cases) in children under 1 year, 12.3% (49 cases) in children aged 1 to less than 3 years, 6.3% (25 cases) in children aged 3 to less than 6 years, 3.8% (15 cases) in children aged 6 to less than 10 years, and 0.7% (3 cases) in children aged 10 years and older. There were no significant changes in gender composition or median age of the *S. aureus*-associated CAP patients during the pandemic compared to 2018 and 2019, as shown in Table 1.

**Table 1:** Comparison of positivity rates, demographic characteristics and co-infection patterns in *S. aureus*-associated patients before and during the COVID-19 pandemic.

Variables	Total	2018	2019	2020	2021	2022
*S. aureus*-positives [No. (%)]	399 (6.7)	97 (6.4)	102 (6.3)	56 (6.4)	78 (7.5)	66 (7.4)
Male patients [No. (%)]	233 (58.4)	52 (53.6)	66 (64.7)	37 (66.1)	42 (53.8)	36 (54.5)
Age (months)	2 (1–9)	2 (1–7)	3 (1–15.25)	2.5 (1–9.75)	2 (1–7)	2 (1–8.25)
Respiratory co-infections [No. (%)]						
* S. pneumoniae*	14 (3.5)	4 (4.1)	4 (3.9)	2 (3.6)	3 (3.8)	1 (1.5)
* H. influenzae*	30 (7.5)	6 (6.2)	17 (16.7)^**a**^	3 (5.4)^**b**^	1 (1.3)^**b**^	3 (4.5)^**b**^
* M. catarrhalis*	36 (9.0)	8 (8.2)	13 (12.7)	3 (5.4)	7 (9.0)	5 (7.6)
* M. pneumoniae*	39 (19.3)	8 (17.0)	16 (21.9)	6 (20.0)	4 (12.1)	5 (26.3)
IVA	2 (0.5)	0 (0.0)	2 (2.0)	0 (0.0)	0 (0.0)	0 (0.0)
IVB	2 (0.5)	0 (0.0)	0 (0.0)	1 (1.8)	1 (1.3)	0 (0.0)
PIV	10 (2.7)	3 (3.2)	1 (1.0)	3 (5.5)	3 (3.9)	0 (0.0)
RSV	44 (11.4)	11 (11.7)	10 (9.9)	6 (10.9)	14 (18.2)	3 (5.0)
ADV	3 (0.8)	1 (1.1)	0 (0.0)	1 (1.8)	0 (0.0)	1 (1.7)

Continuous variable was presented as the median (25–75th percentiles).

^a^*P* < 0.05 Versus 2018.

^b^*P* < 0.05 Versus 2019.

In bacterial co-infections, the rates of *S. aureus*-associated CAP patients co-infected with *S. pneumoniae*, *H. influenzae*, and *M. catarrhalis* were 3.5%, 7.5%, and 9.0%, respectively. Notably, the rate of *H. influenzae* co-infections peaked in 2019 but then declined from 2020 to 2022, showing statistically significant differences (*P* < 0.05). Regarding other pathogens, 19.3% of patients were co-infected with *M. pneumoniae*, and 15.0% had viral co-infections, with RSV being the predominant virus. However, no significant changes were observed in the co-infections of *M. pneumoniae* and viruses during the COVID-19 pandemic compared to the rates in 2018 and 2019, as detailed in Table 1.

### Impact of the COVID-19 pandemic on the drug resistance rates of *S. aureus* and MRSA strains in pediatric CAP patients

As shown in Table 2, the overall resistance rates of *S. aureus* to various antibiotics were: oxacillin (23.1%), penicillin (92.0%), erythromycin (58.6%), clindamycin (51.4%), tetracycline (19.3%), trimethoprim-sulfamethoxazole (11.5%), gentamicin (6.3%), ciprofloxacin (4.5%), levofloxacin (4.5%), and moxifloxacin (3.3%). No strains exhibited resistance to rifampicin, quinupristin-dalfopristin, tigecycline, linezolid, or vancomycin. Significant changes in resistance rates to certain antibiotics were observed during the COVID-19 pandemic. Specifically, the resistance rate to oxacillin significantly increased in 2020 compared to 2018 and 2019. Additionally, the resistance rate to tetracycline from 2020 to 2022 was significantly higher than in 2019. In contrast to 2018, there was a significant decrease in the resistance rates to gentamicin, ciprofloxacin, levofloxacin, and moxifloxacin in 2021. Similarly, 2022 saw significant reductions in the resistance rates to penicillin and gentamicin, all with *P*-values less than 0.05. Resistance rates to other antibiotics did not show significant changes during the pandemic.

**Table 2:** Comparison of resistance rates of *S. aureus* strains before and during the COVID-19 pandemic.

Antibiotics	No. (%) of resistant isolates					
	Total (n = 399)	2018 (n = 97)	2019 (n = 102)	2020 (n = 56)	2021 (n = 78)	2022 (n = 66)
Oxacillin	92 (23.1)	18 (18.6)	20 (19.6)	20 (35.7) ^**a**^^**, b**^	19 (24.4)	15 (22.7)
Penicillin	367 (92.0)	92 (94.8)	96 (94.1)	51 (91.1)	72 (92.3)	56 (84.8) ^**a**^
Erythromycin	234 (58.6)	56 (57.7)	64 (62.7)	33 (58.9)	40 (51.3)	41 (62.1)
Clindamycin	205 (51.4)	46 (47.4)	60 (58.8)	29 (51.8)	35 (44.9)	35 (53.0)
Tetracycline	77 (19.3)	20 (20.6)	11 (10.8)	13 (23.2)^**b**^	18 (23.1)^**b**^	15 (22.7)^**b**^
Trimethoprim-sulfamethoxazole	46 (11.5)	15 (15.5)	14 (13.7)	7 (12.5)	6 (7.7)	4 (6.1)
Gentamicin	25 (6.3)	13 (13.4)	4 (3.9)^**a**^	3 (5.4)	3 (3.8)^**a**^	2 (3.0)^**a**^
Ciprofloxacin	18 (4.5)	6 (6.2)	3 (2.9)	5 (8.9)	0 (0.0)^**a**^	4 (6.1)
Levofloxacin	18 (4.5)	6 (6.2)	3 (2.9)	5 (8.9)	0 (0.0)^**a**^	4 (6.1)
Moxifloxacin	13 (3.3)	6 (6.2)	2 (2.0)	2 (3.6)	0 (0.0)^**a**^	3 (4.5)
Rifampicin	0 (0.0)	0 (0.0)	0 (0.0)	0 (0.0)	0 (0.0)	0 (0.0)
Quinupristin-dalfopristin	0 (0.0)	0 (0.0)	0 (0.0)	0 (0.0)	0 (0.0)	0 (0.0)
Tigecycline	0 (0.0)	0 (0.0)	0 (0.0)	0 (0.0)	0 (0.0)	0 (0.0)
Linezolid	0 (0.0)	0 (0.0)	0 (0.0)	0 (0.0)	0 (0.0)	0 (0.0)
Vancomycin	0 (0.0)	0 (0.0)	0 (0.0)	0 (0.0)	0 (0.0)	0 (0.0)

^a^*P* < 0.05 Versus 2018.

^b^*P* < 0.05 Versus 2019.

All MRSA strains, accounting for 23.1% of the *S. aureus* isolates, were identified by positive results in both oxacillin resistance tests and cefoxitin screening. Aside from showing complete resistance to penicillin, MRSA strains exhibited high resistance rates to erythromycin, clindamycin, and tetracycline. Resistance to trimethoprim-sulfamethoxazole, gentamicin, ciprofloxacin, levofloxacin, and moxifloxacin was less common among these strains. Notably, resistance to erythromycin significantly decreased in 2020 compared to 2018, with a statistical significance (*P* < 0.05). However, resistance rates of MRSA strains to other antibiotics did not show significant changes during the COVID-19 pandemic, as detailed in Table 3.

**Table 3:** Comparison of resistance rates of MRSA strains before and during the COVID-19 pandemic.

Antibiotics	No. (%) of resistant isolates					
	Total (n = 92)	2018 (n = 18)	2019 (n = 20)	2020 (n = 20)	2021 (n = 19)	2022 (n = 15)
Erythromycin	73 (79.3)	17 (94.4)	18 (90.0)	13 (65.0)^**a**^	13 (68.4)	12 (80.0)
Clindamycin	71 (77.2)	16 (88.9)	17 (85.0)	13 (65.0)	13 (68.4)	12 (80.0)
Tetracycline	31 (33.7)	7 (38.9)	4 (20.0)	6 (30.0)	7 (36.8)	7 (46.7)
Trimethoprim-sulfamethoxazole	6 (6.5)	0 (0.0)	1 (5.0)	1 (5.0)	2 (10.5)	2 (13.3)
Gentamicin	1 (1.1)	0 (0.0)	0 (0.0)	0 (0.0)	0 (0.0)	1 (6.7)
Ciprofloxacin	6 (6.5)	1 (5.6)	2 (10.0)	1 (5.0)	0 (0.0)	2 (13.3)
Levofloxacin	6 (6.5)	1 (5.6)	2 (10.0)	1 (5.0)	0 (0.0)	2 (13.3)
Moxifloxacin	3 (3.3)	1 (5.6)	1 (5.0)	0 (0.0)	0 (0.0)	1 (6.7)

^a^*P* < 0.05 Versus 2018.

### Impact of the COVID-19 pandemic on the seasonal patterns of *S. aureus* isolates, viral co-infections, and drug resistant strains

**Figure 1:**
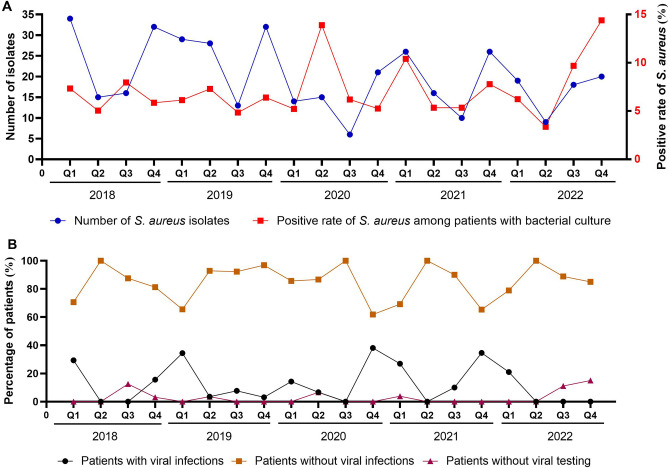
Seasonal patterns of *S. aureus* isolates and viral co-infections from 2018 to 2022. (**A**) Seasonal patterns in the number of *S. aureus* isolates and positive rate among patients with bacterial culture. (**B**) Seasonal patterns in the proportion of *S. aureus*-associated CAP patients with or without viral co-infections, and those without viral testing.

As shown in Fig. 1A, the number of *S. aureus* isolates exhibited seasonal patterns in 2018 and 2019, peaking in the first quarter (Q1, January to March) and fourth quarter (Q4, October to December), and declining in the second quarter (Q2, April to June) and third quarter (Q3, July to September). During the COVID-19 pandemic, these trends remained relatively consistent, with a significant decrease in the first quarter of 2020 and a modest resurgence in the fourth quarter of 2022. The positivity rate of *S. aureus* among pediatric CAP patients showed atypical seasonal trends in 2018 and 2019. After a slight decline in the first quarter of 2020, there was a resurgence in the second quarter. Over the next two years, the peaks of this rate aligned with those of the isolates, reaching the highest level in the fourth quarter of 2022.

Respiratory viruses were tested in up to 97.2% of *S. aureus*-associated CAP patients in our study, allowing analysis of the seasonality of viral co-infections. As depicted in Fig. 1B, the proportion of patients with viral co-infections showed seasonal patterns, peaking in the first or fourth quarter of 2018 and 2021. These peaks were absent in the fourth quarter of 2019 and the first quarter of 2020. Additionally, no resurgence was observed in the fourth quarter of 2022. The trend of patients without viral co-infections generally mirrored the inverse of the trend for viral co-infections across most quarters.

**Figure 2:**
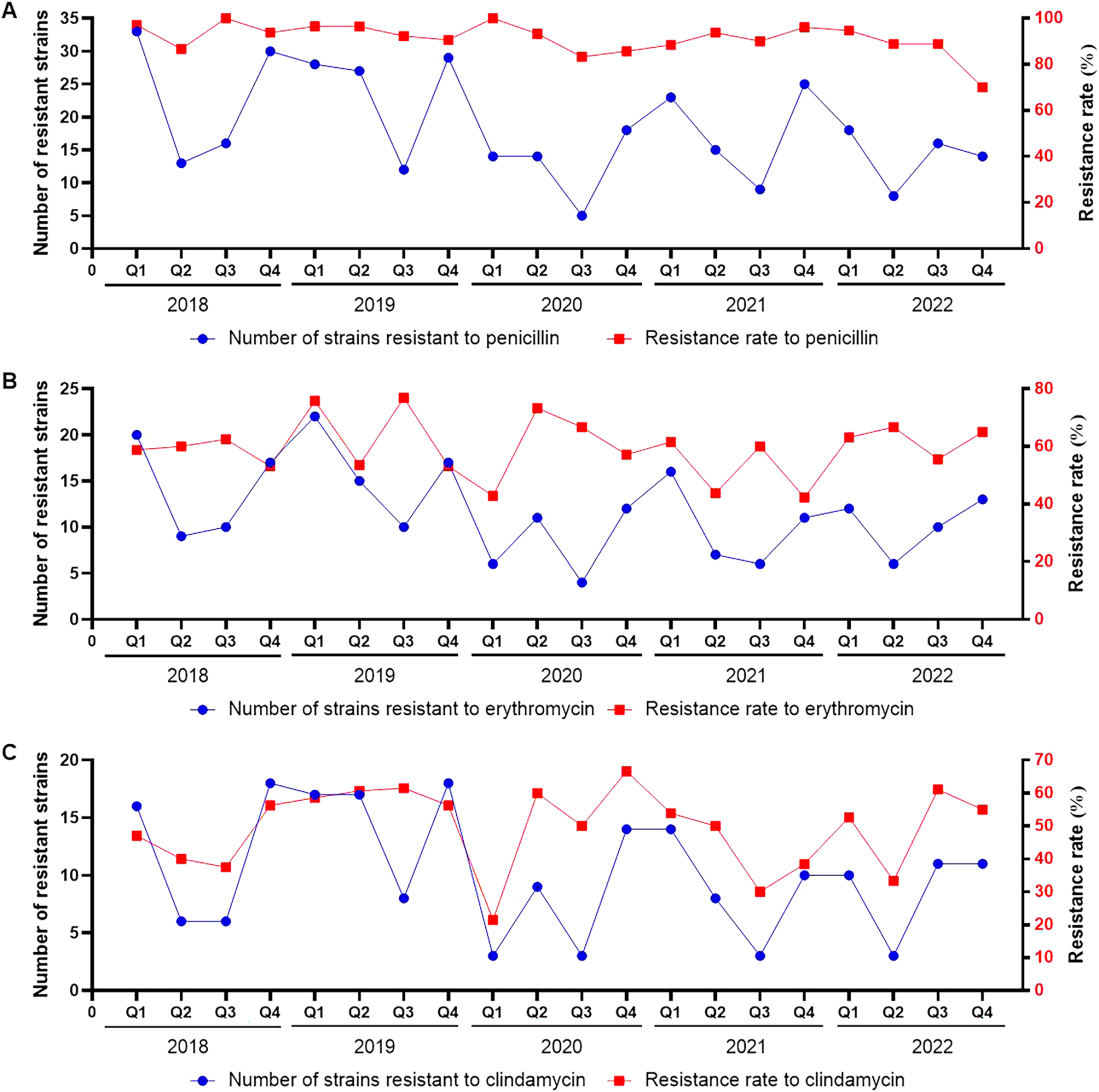
Seasonal patterns in the number and rate of *S. aureus* strains with drug resistance from 2018 to 2022. (**A**) Seasonal patterns in strains with resistance to penicillin. (**B**) Seasonal patterns in strains with resistance to erythromycin. (**C**) Seasonal patterns in strains with resistance to clindamycin.

Seasonal trends were also observed in the prevalence of *S. aureus* strains resistant to penicillin, erythromycin, and clindamycin. The number of resistant strains typically peaked in the first or fourth quarters of 2018 and 2019. During the COVID-19 pandemic, these seasonal patterns shifted, with a decrease in resistant strains in the first quarter of 2020 and the fourth quarter of 2022. In contrast, the seasonal patterns of drug resistance rates were atypical throughout the five-year period, as illustrated in Fig. 2A,B,C.

## Discussion

This study comprehensively analyzed the impact of the COVID-19 pandemic on *S. aureus* infections in children with CAP. The positivity rate, gender composition, and median age of the patients remained relatively stable during the pandemic. Unlike the generally stable co-infections with multiple pathogens, *H. influenzae* isolates significantly decreased during the pandemic compared to 2019. Notably, significant changes were observed in the resistance of *S. aureus* to certain antibiotics, with an increase in resistance to oxacillin and tetracycline, but a decrease in resistance to penicillin, gentamicin, ciprofloxacin, levofloxacin, and moxifloxacin. In MRSA strains, the resistance to erythromycin significantly decreased in 2020.Seasonal variations were noted in the number of *S. aureus* isolates, the proportion of patients with viral co-infections, and the number of strains resistant to specific antibiotics in 2018 and 2019, with peaks in the first or fourth quarter. However, the seasonal patterns in 2020 and 2022 shifted due to local COVID-19 outbreaks, differing from those observed in 2021.

*S. aureus*, commonly found as a colonizer of the nasal microbiota and skin in healthy individuals, can cause infections in community settings^12^. In our study, 6.7% of pediatric CAP patients tested positive for *S. aureus* in bacterial cultures, a rate lower than those reported in other cohorts, which ranged from 8.9 to 12.8%^13,14^. The infection spectrum of many pathogens has significantly changed in recent years due to the COVID-19 pandemic. Consistent with previous findings^15^, the number of *S. aureus* isolates from respiratory specimens in our study markedly decreased between 2020 and 2022. However, no significant changes were observed in the positivity rate of *S. aureus* among patients with bacterial cultures. Similar trends were noted in *S. aureus* isolates from non-respiratory specimens^16–18^. Interestingly, *S. aureus* colonization rates in neonatal intensive care unit patients remained unaffected by visitor restrictions during the COVID-19 pandemic^19^. Additionally, prolonged mask-wearing significantly altered the nasal microbial composition in healthy young adults, with a notable increase in *S. aureus* detected through culture-based analysis^20^. Therefore, the reductions observed in *S. aureus* isolates, which were absent in the positivity rate, might be attributed to the decreased number of children admitted with CAP during the COVID-19 pandemic rather than the direct blocking effects of NPIs during this period.

Consistent with existing literature^21^, our study found that *S. aureus* infections were more frequent in male children of younger age. However, unlike trends in previous studies^13^, we observed a prevalence of *S. aureus* infections particularly in infants. The proportion of males and the median age of *S. aureus*-associated CAP patients did not significantly change during the COVID-19 pandemic, a phenomenon similarly observed in other populations infected with *S. aureus*^9^. Respiratory co-infections have been shown to increase disease severity and complicate effective treatment, underscoring the importance of identifying and managing co-infections^22,23^. In our study, *M. pneumoniae* was the most prevalent pathogen in *S. aureus* co-infections, affecting 19.3% of CAP patients. Additionally, *M. catarrhalis* and RSV emerged as the most common bacterial and viral co-infections, respectively. This highlights the distinct co-infection patterns of *S. aureus* among children in the community setting. During the COVID-19 pandemic, remarkable decreases were observed in respiratory viral and bacterial superinfections in an adult population^24^. Our study assessed the impact of the pandemic on the co-infections of various pathogens in *S. aureus*-associated pediatric CAP patients. Although significant decreases were observed in *H. influenzae* co-infections, the rates of other pathogens did not change significantly during the pandemic, revealing the differential effects of the COVID-19 pandemic.

The AMR of *S. aureus* exacerbates global health risks, leading to increased mortality, prolonged hospital stays, and higher healthcare costs^4,25,26^. Our study revealed high resistance among pediatric CAP patient-derived *S. aureus* strains to penicillins, first-generation macrolides, and lincosamides. Remarkably, no strains resistant to rifampicin, quinupristin-dalfopristin, tigecycline, linezolid, or vancomycin were identified, aligning with similar AMR patterns reported in the literature^27,28^. Notably, the resistance rates to certain antibiotics changed significantly during the COVID-19 pandemic, including increased resistance to oxacillin and tetracycline but decreased resistance to penicillin, gentamicin, ciprofloxacin, levofloxacin, and moxifloxacin. These findings are not entirely consistent with previous studies, which may be attributed to differences in study populations and specimens^11,29–32^. The prevalence of MRSA remains high in recent decades, increasingly appearing in community settings^33^. In our study, MRSA accounted for 23.1% of the *S. aureus* isolates from pediatric CAP patients. The increased MRSA strains during the COVID-19 pandemic in our study, confirmed by the raised resistance to oxacillin, were consistent with most previous findings^34^. However, the resistance rates of MRSA strains to most antibiotics did not change significantly during the pandemic. An exception was the significant decrease in erythromycin resistance observed in 2020. Different drug resistance patterns of MRSA strains during the COVID-19 pandemic have been reported, possibly due to varied sample sources, studied populations, and time periods^35^.

Regarding seasonal patterns, *S. aureus*-associated pneumonia is more common in the cold season^36^. Similarly, the number of *S. aureus* isolates in our study typically peaked in the first or fourth quarter of 2018 and 2019. During the COVID-19 pandemic, the isolates followed a similar trend in 2021. However, the seasonal pattern shifted in 2020 due to a sharp decrease in the first quarter, and in 2022, a non-significant resurgence was observed in the fourth quarter. Notably, these two quarters overlapped with local COVID-19 outbreaks in Chongqing. Shifts in *S. aureus* isolates during the COVID-19 epidemic were also observed in another study^15^. The positivity rate of *S. aureus* fluctuated without clear seasonal patterns in 2018 and 2019. However, a notable resurgence occurred in the second quarter of 2020. Subsequently, the rate peaked in the first or fourth quarter of 2021 and 2022, corresponding with those of the isolates. The rate rebounded to the highest level in the fourth quarter of 2022. The distinct seasonality of the positivity rate during the COVID-19 pandemic might be attributed to decreases in other types of pneumonia, such as those caused by *H. influenzae*. The proportion of viral co-infected patients in 2018 and 2021 demonstrated similar seasonal patterns with *S. aureus* isolates. Yet, an obvious resurgence was absent in the first quarter of 2020 and the fourth quarters of 2019 and 2022, suggesting a potential peak delayed to 2023. The effects of strict NPIs against COVID-19 may have contributed to alterations in the seasonal patterns of viral infections in other Chinese populations as well^37,38^. Typical seasonal patterns were also observed in the number of *S. aureus* isolates resistant to penicillin, erythromycin, and clindamycin in 2018, 2019, and 2021. Notably, the seasonal trends of resistant strains shifted during the first quarter of 2020 and the fourth quarter of 2022. Additionally, the proportion of resistant strains fluctuated without typical seasonal patterns throughout the five years. These findings fill the gap of relevant data during the COVID-19 pandemic and underscore the need for ongoing surveillance in the post-COVID-19 era.

This study acknowledges certain limitations. First, the absence of sputum samples in some children and the potential for nasopharyngeal colonization by *S. aureus* may reduce the accuracy of diagnosing *S. aureus*-associated CAP. Second, the lack of detailed clinical data regarding disease severity, treatment protocols, and patient responses limits our ability to perform a comprehensive analysis of disease dynamics. Third, the *S. aureus* strains analyzed were isolated from a major tertiary hospital, which could introduce survivorship bias, potentially leading to an overestimation of the actual resistance rates of *S. aureus* in broader community settings. Moreover, the retrospective nature of the study may lead to information bias and the single center study could affect the generalizability of the study. The bacterial epidemiology and AMR trends of *S. aureus* observed in our study may not accurately represent those in other geographical areas.

## Conclusions

In conclusion, this study offers comprehensive insights into the impact of the COVID-19 pandemic on the epidemiology and AMR trends of *S. aureus* in pediatric CAP patients. Throughout the COVID-19 pandemic, there were no significant changes observed in the positivity rate of *S. aureus*, nor in the gender composition and age distribution of the patients. Unlike the significant decreases noted in *H. influenzae* co-infections, the prevalence of other respiratory pathogens remained largely unchanged. However, there were notable alterations in the resistance rates of both *S. aureus* and MRSA strains to certain antibiotics. In addition, the typical seasonal patterns in the number of *S. aureus* isolates, the proportion of patients co-infected with viruses, and the number of strains resistant to certain antibiotics shifted to varying degrees. Therefore, a multicenter study involving more participants is essential for continuous surveillance of *S. aureus* infections in the post-COVID-19 era.

## Data Availability

The datasets generated during the current study are available from the corresponding author on reasonable request.
